# Dissonance between posts of health agencies and public comments regarding COVID-19 and vaccination on Facebook in Northern California

**DOI:** 10.1186/s12889-024-20191-8

**Published:** 2024-09-30

**Authors:** Christopher Calabrese, Haoning Xue, Jingwen Zhang

**Affiliations:** 1 Department of Communication, Clemson University, Clemson, SC USA; 2 Department of Communication, University of Utah, Salt Lake City, UT USA; 3 Department of Communication, University of California, Davis, One Shields Avenue, Davis, CA 95616 USA; 4 Department of Public Health Sciences, University of California, Davis, Davis, CA USA

**Keywords:** COVID-19, Vaccination, Health agencies, Unsupervised machine learning, Emotion, Facebook, Social media, Northern California

## Abstract

**Background:**

Public health crises, such as the COVID-19 pandemic, have prompted a need for health agencies to improve their disease preparedness strategies, informing their communities of new information and promoting preventive behaviors to help curb the spread of the virus.

**Methods:**

We ran unsupervised machine learning and emotion analysis, validated with manual coding, on posts of health agencies (*N* = 1588) and their associated public comments (*N* = 7813) during a crucial initial period of the COVID-19 pandemic (January 2020 to February 2021) among nine different counties with a higher proportion of vaccine-hesitant communities in Northern California. In addition, we explored differences in concerns and expressed emotions by two key group-level factors, county-level COVID-19 death rate and political party affiliation.

**Results:**

We consistently find that while health agencies primarily disseminated information about COVID-19 and the vaccine, they failed to address the concerns of their communities as expressed in public comment sections. Topics among public audiences focused on concerns with the COVID-19 vaccine safety and rollout, state mandates, flu vaccination, and frustration with politicians, and they expressed more positive and more negative emotions than health agencies. Further, there were several differences in primary topics and emotions expressed among public audiences by county-level COVID-19 death rate and political party affiliation.

**Conclusion:**

While this research serves as a case study, findings indicate how local health agencies, and their audiences, discuss their perceptions and concerns regarding the COVID-19 pandemic and may inform health communication researchers and practitioners on how to prepare and manage for emerging health crises.

**Supplementary Information:**

The online version contains supplementary material available at 10.1186/s12889-024-20191-8.

## Background

The COVID-19 pandemic is among the ten deadliest pandemics in history. Since the WHO’s pandemic declaration on March 11, 2020, the virus has claimed more than 7 million lives worldwide [1]. As of July 2024, the United States has suffered roughly 17% of the world’s COVID-19 deaths with roughly 1.2 million deaths, while accounting for just over 4% of the world’s population [1]. Further, COVID-19 transmission persists in the US [1], and one in five Americans still view COVID-19 as a significant public health threat [2]. The scale of the virus’ transmission, mutation, and inherent scientific uncertainty have posed an unprecedented challenge to pandemic control and response.

One critical factor for pandemic preparation and response is health communication. COVID-19 is currently the most devastating pandemic in the era of social media, posing many new challenges and conundrums for researchers to rethink what is effective health communication. For example, research discusses the tremendous challenge for individuals in processing the flux of information, including misinformation and uncertain information, and their effects on both physical and psychological health [3]. While the challenge is multifaceted, we can tackle one problem at a time and draw on empirical observations of health communication about COVID-19 on social media to illuminate the causes and potential solutions.

Health agencies have the potential to engage with their audiences on social media. The models of public relations help explain how health agencies may communicate with their audiences [4]. For example, health agencies often use one-way communication, the public information model, to inform and raise awareness [5]. While traditional mass media messaging can disseminate information to the public, social media provide the capability to actively engage in two-way communication with their audiences, providing feedback and responding to concerns surrounding specific issues [3, 4]. While the two-way asymmetrical model focuses on persuading audiences, the two-way symmetrical model aims to achieve mutual agreements which benefit both the organization and the public [4]. Because of the uncertainties and new developments relating to COVID-19, this two-way communication allows health agencies to provide the necessary feedback to their audiences [4]. Dialogic communication focuses on the interaction and relationship between health agencies and their audiences [6], where parties are both able to discuss issues and concerns, while working toward an agreed upon solution. Through mutuality and openness, dialogic communication between an organization and the public is associated with greater trust [7]. In addition, prior work has found that dialogic compared to monologic communication has led to greater social presence, which was associated with increased preventive behavioral intentions [8]. Therefore, during health crises, health organizations may benefit greatly from the use of two-way communication through addressing the concerns of their audiences, while also tailoring their health promotion messaging.

One way to examine the strategies of health agencies’ communication with their audiences is through social media analyses. There are several advantages to examining social media data regarding COVID-19 perceptions. In comparison to the social desirability biases that can be associated with traditional methods such as surveys and interviews, social media provide researchers with unobtrusive behavioral data [3] that can often reveal individuals’ perceptions and views over certain issues [9, 10]. In addition, we can use social media to target specific populations, through keyword searches and geolocation tools, which may be harder to reach in offline contexts. Altogether, the capabilities of social media can provide an overall picture of the beliefs and expressed emotions surrounding health topics among target populations.

While much research has focused on perceptions of COVID-19 at the national or global scale [11, 12], further investigation is necessary to explore the potential impacts of COVID-19 among local communities. The present study examines the posts of health agencies and public comments regarding COVID-19 and vaccination within nine counties in Northern California with a higher prevalence of vaccine-hesitant communities [13] from January 2020 to February 2021. This period was selected as it involved the crucial initial stages of the pandemic over a full year timespan starting in January 2020, when COVID-19 was first determined a public health emergency in the US [14]. The state of California serves as an excellent case study for understanding how health agencies respond and address the COVID-19 crises due to the mandates and preventive measures that took place in the state. For example, in March 2020, Governor Newsom declared a statewide stay-at-home order due to the rising cases of COVID-19 [15]. Though some counties had previously implemented their own mask mandates, a statewide order for mask wearing was issued in June 2020 [16]. In addition, schools were also ordered to shut down in-person learning for the upcoming school year [17]. During the 2020 holiday season, Governor Newsom issued additional masking requirements and restrictions for nonessential businesses [18], as well as a month-long curfew [19], to address increasing COVID-19 cases. Examining how California residents respond to public restrictions and how county-level health agencies respond to public health crises can provide valuable insights for refining communication strategies. This is especially relevant in 2024, as we continue to observe COVID-19 cases and hospitalizations due to new COVID variants [1, 20]. The historical data and trends can inform more effective and targeted public health communication strategies.

We selected nine counties in Northern California (Alpine, Amador, El Dorado, Merced, Nevada, Placer, San Joaquin, Stanislaus, and Yolo) for analyses due to their higher prevalence of vaccine-hesitant communities, including agricultural workers, rural communities, and Slavic communities [13]. Vaccine hesitancy is defined as, “the delay in acceptance or refusal of vaccination despite availability of vaccination services” [21]. These communities have expressed hesitancy toward vaccines and have low vaccination rates compared to the state and the U.S. average [13, 22–24]. Previous research has also found that these target groups have expressed vaccine hesitancy and varying emotions surrounding the HPV vaccine online, including discussions of misinformation [24].

Understanding the role of emotions expressed online is significant, since emotions may play a role in forming and changing our attitudes and behaviors. Similarly, based on emotional contagion theory [25] and affective process theory [26], emotions expressed may diffuse among online audiences, potentially spreading negative emotions regarding COVID-19. Prior research indicates that negative emotional appeals by anti-vaccination movements have contributed to vaccine hesitancy and lower vaccination rates [27, 28]. Further, based on the discrete-emotion approach [29], emotions also have distinct behavioral tendencies; for example, anger is associated with defending one’s own position on an issue, while fear alone is associated with withdrawing or avoiding a threat [29]. In this way, negative emotions expressed in comments may provide indications of vaccine avoidance or hesitancy. One study found that exposure to information about the HPV vaccine on Twitter was associated with state-level vaccination coverage depending on the positive or negative valence of the topics [30]. Similarly, higher emotional intensity has been shown to increase the likelihood of believing fake news [31], where heightened emotions due to the pandemic may prompt individuals to consume COVID-19 misinformation instead of information in support of preventive behaviors [32]. Thus, it is important to preemptively examine these counties’ views toward COVID-19 and the vaccine and understand how their health agencies are addressing the pandemic. Target populations within these nine counties may also have growing concerns over the COVID-19 vaccine due to its perceived quick output and the effects of COVID-19 on work and daily life. We examined the contents and emotions expressed in COVID-19 messaging that health agencies disseminate to their audiences to examine how these agencies respond to public health crises and whether they address the concerns of their communities. Thus, we propose the following research question:

### RQ1

What are the differences in the topics that health agencies and their audiences discuss relating to COVID-19?

To examine the emotions and the topics that emerged from discussions surrounding COVID-19 among health agencies and their audiences, we propose the following research question:

### RQ2

What are the emotions expressed by health agencies and their audiences relating to COVID-19?

Further, as other factors play a role in perceptions surrounding COVID-19 and its related preventive behaviors, we investigate how health agencies differentially engage with their audiences based on county-level COVID-19 death rates and political party affiliation. Partisanship [33–35] and high COVID-19 death rates [36, 37] have been shown to play a role in perceptions about COVID-19 and its related preventive behaviors. These findings will provide a deeper understanding of how local health agencies should tailor their messaging to further promote preventive behaviors during national and international health crises.

Thus, we examined differences among counties with relatively high and low COVID-19 death rates. In response to the COVID-19 death rates within each county, there is a need to push out information and messaging to both promote preventive behaviors and reduce the communities’ negative emotions [27]. Residents living in counties with higher death rates may express more negative emotions, such as anxiety, as the intensity of the COVID-19 pandemic hits their communities. From a theoretical standpoint, behavioral models such as the Extended Parallel Process Model [38] and the Health Belief Model [39] focus on risk and threat perceptions and their impacts on attitudes and behaviors. Addressing the perceived risks of COVID-19 with messages that promote the efficacy of preventive behaviors may improve the adoption of those preventive behaviors like mask-wearing and vaccination [38]. Examining differences between counties with high and low death rates will allow practitioners to view how health agencies are addressing COVID-19, and how the disease may be associated with perceptions of risk by their audiences. We propose the following research question:

### RQ3

How does county-level COVID-19 severity play a role on the topics and emotions expressed related to COVID-19 among health agencies and their audiences?

Further, political party affiliation may play a role in how communities react to public health news and information. As certain policies and mandates take place during times of health crises, COVID-19 may serve as a partisan cue among members of different parties. When faced with a new message, information processing theories such as the Elaboration Likelihood Model [40] and Heuristic Systematic Model [41] indicate that individuals may use cognitive shortcuts like their value predispositions to determine their beliefs about certain science and health subjects [42]. For example, Democrats have shown more support for public restrictions during the pandemic than Republicans [2]. While scientific evidence indicates that preventive methods can help reduce COVID-19 transmission, political partisanship may impede these efforts. This partisan gap provides a stark contrast in the ways in which health agencies should address public health crises, as Republican-leaning counties may express more opposition to COVID-19 mandates and vaccination than Democrat-leaning counties. Understanding how health agencies engage with their audiences of different political leanings may inform practitioners on how to best respond during public health crises. Thus, we propose the final research question:

### RQ4

How does county-level political party affiliation play a role on the topics and emotions expressed related to the COVID-19 among health agencies and their audiences?

## Methods

### Data collection

We scraped Facebook health agency page posts (*N* = 1588) and comments (*N* = 7813) from January 2020 to February 2021. After developing a comprehensive sampling frame of health-related pages located in nine Northern California counties (Alpine, Amador, El Dorado, Merced, Nevada, Placer, San Joaquin, Stanislaus, and Yolo) through geolocation tagging and location-based keyword searches, posts and comments were identified through relevant keyword searches on COVID-19 and the COVID-19 vaccine (see Supplemental Material for the full list of search criteria).

We first identified health-related Facebook pages that used geolocation tagging or a location-based keyword (e.g., Rocklin) and a health agency-related keyword (e.g., health department). The geolocated tags or location-based keywords included both county names and cities (or unincorporated communities) within each county. For health agency keywords, we included terms such as “community clinic” and “health services.” From the keyword search results, we then scraped the pages with Python Selenium [43] package. See the full list of keywords in Table S1.

Based on the resulting list of health-related Facebook pages, we then utilized the CrowdTangle platform [44] to search for posts within each page that contain keywords related to the COVID-19 vaccine (e.g., Covid vaccine). These posts and their associated comments were scraped starting from January 2020 to February 2021 [44]. About 1588 posts with 7813 comments resulted from this comprehensive search. See Table S2 for the number of posts and comments by county.

### Analysis

#### Topic modeling

We utilized Latent Dirichlet Allocation (LDA) [45] topic analysis, a widely used unsupervised machine learning method, to examine the underlying topics expressed in posts and comments. Specifically, we used the LDA model provided by *gensim* for Python [46] to develop a distinctive model and discover topics for all posts, comments, and posts and comments in each group of counties. Each model was developed using all posts or comments in the dataset, with special characters, emojis, and URLs removed. Stop words were removed using the *nltk* library in Python [47]. Each model produced the optimal number of topics based on model perplexity score and coherence to capture model fit and complexity [46], and the weights of keywords that contributed to each topic. We consider a balance between both to make sure the topic model has a good model fit and can produce reliable and interpretable results without being overly complex, since there is no gold standard for estimating. This established method has been widely used to explore a thematic understanding of the online information environment [48].

#### Emotion analysis

Linguistic Inquiry and Word Count (LIWC), a validated software that examines the psycholinguistic properties of words, was used to analyze the specific emotions for posts and comments [49]. This program has been used to examine emotional language related to several topics, including HPV vaccination among online communities [24, 50], breast cancer support forums [51], and general and COVID-19 conspiracy theories on Twitter [52, 53]. We ran LIWC to analyze positive and negative emotions, as well as discrete emotions, including anxiety, anger, and sadness. For each variable, LIWC outputs the percentage of emotion words within a given piece of text, which allows control for word count. Table 1 displays the means and standard deviations for each emotion. Comparisons between posts and comments were conducted utilizing Welch’s t-test for unequal variances, which accounts for unequal sample sizes and variances.

**Table 1:** Means and standard deviations of emotions in health agency posts and audience comments

Emotion	Posts, M(SD)	Comments, M(SD)	Test-statistic	*p*-value
Positive Emotion	2.46 (5.27)	5.67 (14.07)	-15.52	< 0.001
Negative Emotion	0.65 (1.51)	1.92 (7.24)	-14.02	< 0.001
Anxiety	0.24 (0.77)	0.33 (2.89)	-2.37	0.018
Anger	0.19 (1.06)	0.80 (5.46)	-9.03	< 0.001
Sadness	0.09 (0.47)	0.23 (2.12)	-5.23	< 0.001

Note. Test statistic is a t-statistic based on Welch’s t-tests for unequal variances

#### Robustness check

To add an additional layer of analysis and validate the topic modeling and emotion analysis findings, the full sample of posts (*N* = 1588) and a random sample of comments (*N* = 1565; 20%) were manually coded. Three coders went through two rounds of coding to achieve acceptable reliability before independently coding a set of posts and comments. To estimate reliability, coders independently categorized a random sample of posts, and the resulting reliability between coders was acceptable (α = 0.65-0.98). Details on the codebook, coding process, and individual intercoder reliabilities can be found in the Supplemental Materials.

#### County comparisons

To examine differences between county-level characteristics, we conducted the same procedures for topic modeling and emotion analyses. Comparisons of expressed emotion were conducted between county-level COVID-19 death rates and political party leaning for both posts and comments (Tables 2 and 3).

##### County-level COVID-19 death rates

We assessed differences between counties with high and low COVID-19 death rates through data collected by state and local health agencies^1^; we examined the rates in March 2021 to match the time period of our dataset. To account for population differences, we compared counties by COVID-19 deaths per 100,000 people. Counties with more than 100 COVID-19 deaths per 100,000 were categorized as “high”, resulting in four counties with high death rates, and five counties with lower death rates. The researchers chose this categorization criterion due to the clear cutoff points found in the reported state and local health data, as well as the nature of the disease.

**Table 2:** Means and standard deviations of emotions by counties with low versus high COVID-19 death rates

	Low Death Rate		High Death Rate	
Emotion	Posts M(SD)	Comments M(SD)	Posts M(SD)	Comments M(SD)
Positive Emotion	2.71 (4.67)	7.05 (16.00)	2.33 (5.55)	4.42 (11.92)
Negative Emotion	0.63 (1.44)	2.00 (6.72)	0.67 (1.54)	1.85 (7.67)
Anxiety	0.26 (1.00)	0.41 (3.49)	0.23 (0.62)	0.26 (2.21)
Anger	0.17 (0.74)	0.61 (4.07)	0.20 (1.20)	0.97 (6.46)
Sadness	0.13 (0.67)	0.31 (2.38)	0.08 (0.31)	0.17 (1.85)

##### County-level partisanship

To examine how political party leaning may play a role in discussions surrounding COVID-19, we compared emotions expressed and discussions between Democrat and Republican-leaning counties. Counties were categorized using the California Secretary of State voter registration database for the 2020 presidential election^2^. Each county was labeled with the political party that had the highest percentage of registered voters, resulting in six Democrat-leaning counties and three Republican-leaning counties.

**Table 3:** Means and standard deviations of emotions by Republican versus Democrat-leaning counties

	Republican-Leaning		Democrat-Leaning	
Emotion	Posts M(SD)	Comments M(SD)	Posts M(SD)	Comments M(SD)
Positive Emotion	2.64 (4.54)	6.53 (15.93)	2.39 (5.53)	5.22 (12.97)
Negative Emotion	0.60 (1.22)	2.14 (7.00)	0.67 (1.61)	1.80 (7.35)
Anxiety	0.21 (0.64)	0.49 (4.05)	0.25 (0.81)	0.25 (2.03)
Anger	0.16 (0.61)	0.63 (3.96)	0.20 (1.19)	0.89 (6.10)
Sadness	0.14 (0.66)	0.34 (2.61)	0.08 (0.36)	0.18 (1.80)

## Results

### Health agency posts versus audience comments

To answer RQ1, we qualitatively assessed the topic modeling results for both overall health agency posts and audience comments. Four topics emerged among health agency posts, all relating to disseminating information (see Table 4). The four topics include: health information related to Stanislaus County^3^, health information and resources about getting the COVID-19 vaccine, community resources for COVID-19, and information about flu vaccination. However, audience comments expressed opinions on more diverse aspects of daily life related to the pandemic and the COVID-19 vaccine. Seven topics emerged focusing on opinions toward mask-wearing, frustrations with politicians, good news for workers relating to the COVID-19 vaccine, opinions toward state work-at-home orders and mandates, expressing thanks for the COVID-19 vaccine, opinions over one’s rights to get the COVID-19 vaccine, and opinions over getting the flu shot. These topics in posts and comments have been validated with manual coding, where we found posts were dominated by county-specific resources and vaccine-related discussions (Table 5), whereas comments revolved around political conversations and vaccine safety (Table 6).

**Table 4:** Topics and keywords for overall health agency posts and audience comments

Type	Topic	Keywords
Posts	1. Health information specific to Stanislaus County.	*vaccine*,* covid*,* vaccines*,* modesto*,* la*,* stanislaus*,* dose*,* coronavirus*,* clinics*,* county*
	2. Health information and resources for the COVID-19 vaccine.	*covid*,* vaccine*,* vaccines*,* cdc*,* gov*,* coronavirus*,* get*,* learn*,* ncov*,* benefit*
	3. COVID-19 for community health.	*covid*,* vaccine*,* health*,* county*,* public*,* state*,* care*,* receive*,* community*,* include*
	4. Information for getting the flu vaccine.	*covid*,* vaccine*,* flu*,* coronavirus*,* county*,* merced*,* health*,* question*,* live*,* facebook*
Comments	1. Opinions surrounding mask wearing.	*People*,* mask*,* vaccine*,* like*,* wear*,* know*,* don’t*,* need*,* die*,* live*
	2. Frustration with politicians.	*get*,* vaccine*,* trump*,* say*,* release*,* yes*,* like*,* want*,* work*,* job*
	3. Good news for people who work.	*good*,* news*,* go*,* work*,* awesome*,* think*,* order*,* melissa*,* vaccine*,* check*
	4. Opinions surrounding state mandates and compliance.	*covid*,* order*,* notice*,* stop*,* comply*,* bar*,* work*,* help*,* personal*,* direct*
	5. Expressing thanks.	*vaccine*,* thank*,* covid*,* people*,* time*,* effect*,* nope*,* family*,* long*,* know*
	6. Opinions over right to get the vaccine.	*vaccine*,* vaccinate*,* right*,* people*,* risk*,* health*,* home*,* like*,* county*,* covid*
	7. Opinions over getting the flu vaccine.	*flu*,* county*,* shoot*,* vaccine*,* get*,* wait*,* dose*,* covid*,* need*,* people*

To answer RQ2, we examined differences between posts and comments by the emotions expressed in each message. Overall, audiences expressed more positive emotion (t(6672.31) = -15.52, *p* < .001), negative emotion (t(9395.21) = -14.02, *p* < .001), anxiety (t(8894.52) = -2.37, *p* = .018), anger (t(9389.89) = -9.03, *p* < .001), and sadness (t(9360.44) = -5.23, *p* < .001) than the health agencies. Similar to the contents discussed, audience comments also had a diverse range of expressed emotions compared to health agencies.

**Table 5:** Frequency of emotional valence and topics in coded posts (*N* = 1588)

Variables	*N* (%)
**Emotional Valence**	
Positive	105 (6.6%)
Neutral	1461 (92.0%)
Negative	19 (1.2%)
**Topics**	
County-specific	596 (37.5%)
Disease-specific	114 (7.2%)
Vaccine-specific	1088 (68.5%)
State mandates	90 (5.7%)
Flu vaccine	157 (9.9%)
Government resources	133 (7.1%)

Note. Coding categories were not mutually exclusive

**Table 6:** Frequency of emotional valence and topics in coded comments (*N* = 1565)

Variables	*N* (%)
**Emotional Valence**	
Positive	199 (12.7%)
Neutral	749 (47.9%)
Negative	479 (30.6%)
**Topics**	
Vaccine safety	134 (8.6%)
Political discussions	156 (10.0%)
Mask wearing	21 (1.3%)
State mandates	65 (4.2%)
Vaccine rollout	123 (7.9%)
Flu vaccine	45 (2.9%)
Sheriff enforcement	20 (1.3%)
Expressing thanks	99 (6.3%)

Note. Coding categories were not mutually exclusive

### Differences by county-level COVID-19 death rates

To answer RQ3, we examined the differences in topics and emotions between posts and comments from counties with high versus low COVID-19 death rates.

#### Health agency posts

Among high COVID-19 death county posts, three topics emerged focusing on information in Stanislaus County, messages involving a Facebook Live health information series by Merced County Public Health, and information and resources about COVID-19. For low COVID-19 deaths county posts, four topics resulted centering around issues with COVID-19 test scammers, flu vaccination, government resources for COVID-19, and county-level updates for the COVID-19 vaccine. Health agencies in high-death rate counties discussed more COVID-19 and vaccine-specific information, information about state mandates and government resources (Table S8).

Emotion analysis found no differences in any emotion between high versus low COVID-19 death rate counties; however, manual coding indicated that low-death rate posts expressed more positive words compared to high-death rate posts (see Table S8).

#### Audience comments

Among high death rate county comments, four topics emerged focusing on opinions about the vaccine rollout, prevention methods (mask wearing and flu vaccine), work-at-home and other state mandates, and expressing desires to open businesses. However, among low death rate counties, five topics focused on expressing thanks for COVID-19 vaccines and testing, opinions about vaccines for work, expressing frustration over the judge’s ruling to release prisoners due to COVID-19, thanking for COVID-19 vaccine information, and questions and concerns on how high-risk individuals deal with COVID-19. Manual coding indicated that audiences in high-death rate counties discussed state mandates more but expressed thanks to others less than low-death rate counties (see Table S10).

Low-death rate counties expressed more positive emotion (*t*(6813.31) = 8.16, *p* < .001), anxiety (*t*(6162.91) = 2.33, *p* = .020), and sadness (*t*(6988.49) = 2.98, *p* = .003) compared to comments in high-death rate counties. Further, high-death counties’ comments expressed more anger (*t*(6999.83) = -2.97, *p* = .003). In addition, manual coding found that audiences in high-death rate counties expressed less positive emotion and more negative emotion than audiences in low-death rate counties (see Table S10).

### Differences by county-level political party affiliation

To answer RQ4, we examined the differences in topics and emotions expressed between posts and comments from Republican versus Democrat-leaning counties.

#### Health agencies posts

Health agencies in Republican-leaning counties focused on information about COVID-19 cases, COVID-19 vaccine information and resources, flu vaccine recommendations, and COVID-19 vaccine resources. In Democrat-leaning counties, posts focused on two topics: information about the COVID-19 vaccine in Stanislaus and county and government resources for the COVID-19 vaccine. Manual coding revealed that Democrat-leaning counties discussed information about COVID-19, state mandates, and government resources more than Republican-leaning counties (Table S9). There were also no significant differences in emotions expressed between Republican and Democrat-leaning counties in both emotion analysis and manual coding results.

#### Audience comments

Audience comments in Republican-leaning counties focused on four topics, expressing thanks for COVID-19 information, questions about the COVID-19 vaccine, concerns surrounding the release of prisoners due to COVID-19, and discussions about receiving confirmation emails for vaccines or testing. However, in Democrat-leaning counties, five topics focused on opinions toward (1) flu shot recommendations, (2) state mandates, (3) enforcement of the stay-at-home orders in Stanislaus, and (4) expressing thanks and (5) discussions about Trump and the COVID-19 vaccine. Manual coding revealed that Republican-leaning counties’ audiences had more expressions of thanks and political discussions, but fewer discussions of state mandates (see Table S11).

Among audience comments, Republican-leaning counties expressed more positive emotion (*t*(4557.44) = 3.67, *p* < .001), as revealed by both emotion analysis and manual coding (Table S11). In addition, Republican-leaning counties expressed more negative emotion (*t*(5660.72) = 1.99, *p* = .046), anxiety (*t*(3394.07) = 2.86, *p* = .004), and sadness (*t*(4035.80) = 2.93, *p* = .003), while Democrat-leaning counties expressed more anger (*t*(7449.69) = -2.29, *p* = .022).

## Discussion

Our study examined the dissonance between posts of health agencies and public comments regarding COVID-19 and vaccination on Facebook in Northern California. Overall, we observed a significant gap in how health agencies address public concerns. Health agencies primarily adopted traditional one-way communication to disseminate health information and community-relevant resources, which reflects the public information model [4]. This falls in line with prior research that found health organizations, as well as nonprofits, tend to mainly focus on sharing information [5, 24, 54, 55]. In contrast, audiences expressed a variety of emotions (both positive and negative) in the comments compared to the health agencies, displaying active engagement and concerns with COVID-19-related issues that may directly affect them. These issues include their opinions about masks, stay-at-home orders and mandates, their rights, and the flu vaccine, as well as their frustration with politicians. This discrepancy between health promotion efforts and the public’s feedback highlights a critical need for local public health agencies to not only guide local audiences to community resources but also adopt more responsive and interactive two-way communication strategies to actively address their concerns and effectively facilitate health behavior change [4].

While it is important for health agencies to disseminate necessary information to their audiences, especially since communicating about vaccination and COVID-19-related policies are shown to promote vaccination [56], there is a noticeable misalignment between their priorities and the priorities of their audiences. This finding is not surprising, as agencies often focus on distributing critical health information and resources and avoid potentially losing control when engaging in more dialogic communication with their audiences [5, 57]. For example, if an individual with strongly held COVID-19 conspiracy beliefs decides to argue with a post, the health agency would need to consider several implications of addressing the individual, including whether the information shared in the interaction is accurate, how other audience members would perceive and interpret the information shared, as well as how the audience would perceive the health organization itself. It is likely that interacting with audiences may be perceived as too risky and unpredictable for health agencies [57].

However with less than 30% of US adults indicating they had “a great deal” of trust in local public health departments as a health information source [58], the lack of open dialogue also suggests a missing opportunity for health promotion, where agencies can engage with their audiences and develop more tailored messages that can address the concerns of their audiences, while still encouraging the importance of preventive health behaviors [59]. For example, audience comments expressed more negative emotions than health agencies’ posts. Addressing these anxieties and fears through tailored messages on recommended preventive actions could improve self-efficacy among their audiences [38]. Similarly, providing clear explanations for COVID-19-related restrictions and mandates may help alleviate the audiences’ anxieties with governmental restrictions and make the public feel heard and validated. As researchers conduct additional studies on addressing an emerging crisis, such as with COVID-19, recommendations may change or adjust over time. Health agencies should provide clear updates on new recommendations based on the most up-to-date findings. Further, emphasizing the health and social benefits of policies such as vaccine mandates, can help shift the audiences’ attention and attitudes toward these actions [42]. As an emerging crisis evolves with new information flowing each day, health agencies may need to switch their focus from merely spreading awareness, one-way communication, to crafting more tailored, persuasive messaging to address needs and concerns while building trust with their audiences. We further illustrate this point by discussing two key factors that influence audience concerns: county-level death rates and party affiliation.

### County-level COVID-19 death rates

Health agency posts in high-death counties did seem to be more tailored than in low-death counties as an effort to encourage COVID-19 preventive behaviors. For example, one topic that emerged centered around promoting a Facebook Live event by the Merced County Department of Public Health. It is worth noting that the health program occurred more than once and involved panelists from different cultural backgrounds. With information in Spanish, this event provided health information to specific target audiences in the region, indicating targeted efforts among health agencies to reduce cases of COVID-19 within their communities. These findings may indicate that health agencies in high COVID-19 death rate counties are likely to incorporate more tailored messaging to address the already higher death rates in their areas. However, we express caution with this conclusion since we find this evidence from two individual cases, as we observed tailored messages from health agencies in Merced and Stanislaus. Regardless, these cases may still serve as models to other counties with higher COVID-19 death rates for how to develop more tailored messaging. On the contrary, health agencies in counties with low COVID-19 death rates adopted a more general approach by focusing on broad issues, such as scammers that lie about providing COVID-19 tests and information on flu vaccination and government resources for COVID-19.

When comparing differences between audience comments, audiences in high-death counties are less focused on COVID-19’s health implications and are more concerned about the impacts on their daily lives, with high levels of anger, especially regarding their freedoms on vaccine rollout, work-at-home orders, preventive methods, and the re-opening of businesses. Because of these concerns, health agencies in these areas need to develop messaging that not only alleviates their audience’s anger, but also redirects their attention toward the health implications of the COVID-19 pandemic to encourage vaccine uptake.

Audiences in low COVID-19 death rate counties primarily focused on the health aspect of COVID-19, such as expressing thanks for COVID-19 information and vaccines, and questions about handling higher-risk individuals. It may be inferred that audiences already have a baseline acceptance of COVID-19 and its related measures. Rather, they are primarily concerned with protecting others, including higher-risk individuals and people going back to work, as well as protecting against the potential implications of prisoner release. Because these audiences have a sense of acceptance toward COVID-19 preventive behaviors, health agencies may prefer to develop messages about protecting specific populations and informing their audiences about related issues due to the pandemic.

### County-level political party affiliation

Health agency posts from both Republican and Democrat-leaning counties primarily focused on providing information and resources related to COVID-19 with similar expressed emotions, though the Democrat-leaning counties were more likely to provide information about COVID-19, mandates, and government resources compared to Republican-leaning counties. These findings fall in line with our overall results that health agencies are primarily focused on disseminating information rather than providing tailored communication that addresses the health-related concerns of their audiences. In addition, health agencies may want to maintain trust with their community and avoid potentially seeming biased, especially with a politicized issue like COVID-19, which may indicate why they prefer traditional one-way communication with their audiences.

Among comments, Republican audiences discussed more political issues related to COVID-19 with heightened emotions compared with Democrat audiences, although discussions surrounding Trump were highly salient among Democrat counties. These findings highlight a need for health agencies to acknowledge audience concerns regardless of political leaning, including concerns over individual liberties and the uncertainties revolving around an emerging crisis. Given the politicization of COVID-19, providing health information alone may not be enough to effectively address and engage communities. Instead, there is a pressing need for tailored persuasive messaging to redirect audiences’ attention toward the health consequences of COVID-19 and resonate with diverse audience perspectives.

### Limitations and future directions

This study is not without limitations. First, we examined Facebook pages which provided an understanding of how specific geo-located Northern California health agencies disseminate information to their audiences. If provided access to social media platforms, future research should examine other social media platforms in which health agencies communicate with their audiences, such as Instagram or X (formerly known as Twitter). Further, to address communities without access to health agencies in their local areas, future research can examine Facebook groups and other more private platforms to gauge perceptions of COVID-19, specifically among high-risk target populations. Second, our study examined discussions surrounding COVID-19 over thirteen months during a crucial period of the pandemic. As new information about COVID-19 is discovered and shared with the public, such as news about the 2023–2024 COVID-19 updated vaccine, further investigation may be necessary to examine how communities’ perceptions and local health agencies’ strategies change over time. Further, while our study focused on interactions between health agencies and their audiences, future work could examine interactions between audience members surrounding health topics like COVID-19. Although we collected unobtrusive social media data, which has advantages compared to self-report surveys and interviews, a small portion of online users may not post content that expresses their true beliefs regarding health issues. Similarly, we did not distinguish whether some of the audience comments were bot-generated; while outside the scope of this study, this may be an important avenue for future research. Despite these limitations, this research serves as a case study for how community health agencies and their audiences discuss their concerns regarding the COVID-19 pandemic and examines the role of important factors, partisanship and death rates, which informs health communication researchers and practitioners on how to best prepare and manage for emerging health crises.

## Conclusion

Our research serves as a case study for how local health agencies and their audiences discuss their perceptions surrounding the COVID-19 pandemic. Public health professionals should develop message strategies to alleviate the anxiety and other negative emotions that communities express about COVID-19. Further, health agencies should engage in two-way communication with their audiences to address the communities’ concerns, especially regarding health orders and recommendations. Lastly, as we prepare for future pandemics, local health agencies should be cognizant of the potential role of partisanship and local death rates, as these county-level differences may require more tailored communication strategies to address their audiences’ concerns and promote preventive health behaviors.

## Electronic supplementary material

Below is the link to the electronic supplementary material.

Supplementary Material 1

## Data Availability

The datasets used and/or analyzed during the current study are available from the corresponding author on reasonable request.

## Footnotes

1 This information was last accessed on May 8th, 2021, can be found at the New York Times (https://www.nytimes.com/interactive/2021/us/california-covid-cases.html).

2 This information was last accessed on May 8th, 2021, and can be found at the California Secretary of State website (https://www.sos.ca.gov/elections/voter-registration/voter-registration-statistics).

3 A considerable number of posts were found in Stanislaus County (see Table S2). This may be due to multiple factors, including the higher COVID-19 death rate in the county, as well as the online presence of health agencies in the county overall.

## Abbreviations

COVID-19: Coronavirus disease
HPV: Human papillomavirus
WHO: World Health Organization
