# Supplementary material for: Dissonance between posts of health agencies and public comments regarding COVID-19 and vaccination on Facebook in Northern California
Source: BMC Public Health. 2024 Sep 30;24:2672. doi: 10.1186/s12889-024-20191-8 (PMC11441105; doi:10.1186/s12889-024-20191-8)
Supplement: Supplementary file 1 — Supplementary Material 1 [file 12889_2024_20191_MOESM1_ESM.docx]

# Supplementary Material

## Search Criteria

Facebook Page Search Term Criteria

Table S1. List of Location-Based Keywords and Health Agency Search Terms

Search Terms to Gather COVID-19 Related Posts

## County Information

Table S2. Frequencies of Posts and Comments by County

Table S3. Categorization of Counties by Political Party Leaning and COVID-19 Death Rates

**Topic Modeling Results**

Table S4. Topic Results for Posts and Comments among Counties with High Death Rates

Table S5. Topic Results for Posts and Comments among Counties with Low Death Rates

Table S6. Topic Results for Posts and Comments among Republican-Leaning Counties

Table S7. Topic Results for Posts and Comments among Democrat-Leaning Counties

## Coding Analyses

Table S8. Frequency of Emotional Valence and Topics in Posts by County-Level Death Rate

Table S9. Frequency of Emotional Valence and Topics in Posts by Political Party Affiliation

Table S10. Frequency of Emotional Valence and Topics in Comments by County-Level Death Rate

Table S11. Frequency of Emotional Valence and Topics in Comments by Political Party Affiliation

## Facebook Page Search Term Criteria

To gather relevant Facebook pages among the nine counties, the following search term criteria were utilized (See Table S1):

- A location-based keyword OR geo-location tag that either included the county name or the cities (or unincorporated communities) within each county, e.g., Rocklin, AND
- A health agency-related keyword, e.g., Health department.

### **Table S1**

### List of Location-Based Keywords and Health Agency Search Terms

| Location-based Keywords | Health Agency Keywords |
| --- | --- |
| Alpine (12 search terms):  Alpine, Alpine Village, Bear Valley, Fredericksburg, Kirkwood, Lake Alpine, Loope, Markleeville, Mesa Vista, Paynesville, Sorensens, Woodfords  Amador (6 search terms):  Amador, Amador City, Ione, Jackson, Plymouth, Sutter Creek  El Dorado (3 search terms):  El Dorado, Placerville, South Lake Tahoe  Merced (7 search terms):  Merced, Atwater, Dos Palos, Gustine, Livingston, Los Banos, Merced  Nevada (3 search terms):  Nevada, Grass Valley, Nevada City  Placer (6 search terms):  Placer, Auburn, Colfax, Lincoln, Rocklin, Roseville  San Joaquin (8 search terms):  San Joaquin, Escalon, Lathrop, Lodi, Manteca, Ripon, Stockton, Tracy  Stanislaus (10 search terms):  Stanislaus, Ceres, Hughson, Modesto, Newman, Oakdale, Patterson, Riverbank, Turlock, Waterford  Yolo (5 search terms):  Yolo, Davis, West Sacramento, Winters, Woodland | Community clinic  Health  Health center  Health department  Health services  Healthcare  Hospital  Public health |

## Search Terms to Gather COVID-19 Related Posts

Coronavirus, COVID-19, COVID, Corona, Johnson and Johnson, Moderna, Pfizer.

## County Page Information

### **Table S2**

### Frequencies of Posts and Comments by County

| **County** | **Posts, N** | **Comments, N** |
| --- | --- | --- |
| Alpine | 2 | 12 |
| Amador | 63 | 5 |
| El Dorado | 167 | 1382 |
| Merced | 277 | 815 |
| Nevada | 98 | 879 |
| Placer | 217 | 1289 |
| San Joaquin | 214 | 301 |
| Stanislaus | 488 | 2983 |
| Yolo | 62 | 147 |

### **Table S3** Categorization of Counties by Political Party Leaning and COVID-19 Death Rates

| **County** | **Political party leaning by  registered voters** ^a^ | **COVID-19 deaths  per 100,000** ^b^ |
| --- | --- | --- |
| Alpine | Democrat | Low |
| Amador | Republican | High |
| El Dorado | Republican | Low |
| Merced | Democrat | High |
| Nevada | Democrat | Low |
| Placer | Republican | Low |
| San Joaquin | Democrat | High |
| Stanislaus | Democrat | High |
| Yolo | Democrat | Low |

*Note.* ^a^ Political party leaning was assessed by the political party with the highest percentage of registered votes for the 2020 presidential election. This information was accessed as of May 8^th^, 2021 and can be found at the California Secretary of State website (<https://www.sos.ca.gov/elections/voter-registration/voter-registration-statistics>).

^b^ COVID-19 deaths per 100,000 were assessed through state and local health agencies and can be found at the New York Times (<https://www.nytimes.com/interactive/2021/us/california-covid-cases.html>); the information has been accessed as of May 8^th^, 2021. Counties with more than 100 COVID-19 deaths per 100,000 were categorized as ‘High.”

## Topic Modeling Results

### **Table S4**

### Topic Results for Posts and Comments among Counties with High COVID-19 Death Rates

| **Type** | **Topic** | **Keywords** |
| --- | --- | --- |
| Posts | 1. Stanislaus County-specific information. | *vaccine, covid, county, health, stanislaus, public, coronavirus, dose, modesto, vaccines* |
|  | 2. Information related to a Facebook Live series on health in English and Spanish. | *Covid, flu, la, health, facebook, live, dr, question, el, nuestra* |
|  | 3. Informational government resources for COVID-19. | *covid, vaccine, vaccines, cdc, coronavirus, gov, learn, get, la, pandemic* |
| Comments | 1. Opinions about the vaccine rollout. | *people, vaccine, like, need, line, care, think, home, thank, come* |
|  | 2. Opinions on prevention methods (mask-wearing and flu vaccine). | *vaccine, flu, get, covid, mask, shoot, people, wear, trump, time.* |
|  | 3. Opinions on work-at-home and other state mandates. | *work, bar, order, comply, covid, help, thank, spread, gym, personal* |
|  | 4. Expressing desires to open businesses. | *county, people, go, right, want, stanislaus, know, business, open, need* |

### **Table S5**

### Topic Results for Posts and Comments among Counties with Low COVID-19 Death Rates

| **Type** | **Topic** | **Keywords** |
| --- | --- | --- |
| Posts | Information to report scammers relating to COVID-19 tests. | *scammers, covid, school, scam, fake, charities, charitywatch, charity, test, check.* |
|  | Information for the flu shot. | *flu, covid, vaccine, placer, shoot, county, health, vaccines, coronavirus, gov* |
|  | Government and county resources for COVID-19 vaccine information. | *vaccine, covid, health, county, public, page, government, aspx, new, edcgov* |
|  | New information and updates about the COVID-19 vaccines. | *vaccine, covid, county, health, placer, update, information, receive, nevada, dose* |
| Comments | Expressing thanks for COVID-19 vaccines and testing. | *vaccine, thank, like, think, news, test, trust, yes, vaccines, right* |
|  | Opinions about vaccines for work and jobs. | *vaccine, get, county, people, work, im, vaccines, time, dose, covid* |
|  | Expressing frustration over judge ruling to release prisoners due to COVID-19. | *release, like, people, state, think, judge, people, ed, robinson, lol* |
|  | Thanking for COVID-19 vaccine information. | *thank, vaccine, information, good, marie, mask, post, know, get, covid* |
|  | Questions and concerns on how high-risk individuals deal with COVID-19. | *vaccine, people, covid, mask, go, death, risk, health, virus, die* |

### **Table S6**

### Topic Results for Posts and Comments among Republican-Leaning Counties

| **Type** | **Topic** | **Keywords** |
| --- | --- | --- |
| Posts | 1. COVID-19 case information. | *covid, vaccine, county, placer, amador, health, public, case, age, include* |
|  | 2. COVID-19 vaccine information and resources. | *vaccine, covid, health, dose, workers, county, page, care, government, aspx* |
|  | 3. Recommendation for the flu vaccine. | *flu, covid, shoot, season, important, protect, get, bite, vaccine* |
|  | 4. COVID-19 vaccine resources. | *vaccine, covid, vaccines, learn, facebook, health, gov, vaccination, get* |
| Comments | 1. Expressing thanks for COVID-19 related information. | *thank, post, vaccine, county, people, think, like, information, link, vaccines* |
|  | 2. Questions surrounding the COVID-19 vaccine. | *vaccine, covid, dose, people, need, health, vaccines, get, know, death* |
|  | 3. Issues surrounding the release of prisoners due to COVID-19. | *People, covid, get, release, thank, vaccine, judge, good, mask, know* |
|  | 4. Discussions on receiving confirmation emails for vaccines or testing. | *vaccine, know, make, health, people, public, effect, die, email, time* |

### **Table S7**

### Topic Results for Posts and Comments among Democrat-Leaning Counties

| **Type** | **Topic** | **Keywords** |
| --- | --- | --- |
| Posts | 1. Stanislaus COVID-19 vaccine information. | *vaccine, county, health, stanislaus, covid, modesto, public, dose, vaccines, california* |
|  | 2. County and government resources for the COVID-19 vaccine. | *covid, vaccine, vaccines, coronavirus, health, county, gov, merced, cdc, la* |
| Comments | 1. Opinions toward flu shot recommendations. | *flu, people, like, right, want, say, get, shoot, time, business* |
|  | 2. Opinions on state mandates. | *work, covid, help, bar, comply, spread, gym, order, stop, notice* |
|  | 3. Expressing thanks. | *vaccine, thank, people, get, covid, need, care, know, mask, im* |
|  | 4. Opinions on the enforcement of the stay-at-home orders in Stanislaus. | *county, stanislaus, page, sign, sheriff, essential, website, need, work, department* |
|  | 5. Discussions about Trump and the COVID-19 vaccine. | *vaccine, vaccines, covid, dont, people, trump, want, nope, virus, news* |

## Coding Analyses

## Codebook for Topic Analysis

We utilized the following codebook to validate our topic modeling results. To ensure objectivity, we did not code for discrete emotions expressed in text (such as anxiety or sadness). Coding categories were not mutually exclusive.

**Emotional valence:** Positive, Neutral, or Negative

**Content type:**

***Posts***

- County specific information
  - Where to get the vaccine in the county, community health
- COVID-19 (disease-specific) information
  - New information, cases, etc.
- COVID-19 (vaccine) information
  - Information specific to vaccines.
- COVID-19 (state mandates) information
  - Mask wearing, stay-at-home order, etc.
- Flu vaccine information
  - Recommendation to get the flu vaccine during the COVID-19 pandemic.
- Governmental information resources
  - National level government references, links to CDC, etc.

***Comments***

- Political discussion (Trump, release prisoners, etc.)
- Opinions of vaccine safety/effectiveness.^*^
- Opinions on mask wearing.
- Opinions on state mandates (not related to mask wearing).
- Opinions on vaccine rollout phases (getting in line and waiting for vaccine, high risk individuals expressing need for vaccine, essential workers expressing need, etc.)
- Opinions over the flu vaccine.
- Opinions of Sheriff’s enforcement of stay-at-home orders.
- Expressing thanks.

^*^ Because vaccines were mentioned in every topic in the comments section, we included vaccine safety/effectiveness as a separate topic.

## Coding Reliabilities

To validate our topic modeling results, we also manually coded the full sample of posts (N=1588) and a random sample of comments (N=1565; 20%). To estimate reliability, coders independently categorized a random sample of posts (N=125) and comments (N=131). The resulting reliability between coders was acceptable.

**Posts:**

Positive = .78

Neutral = .96

Negative = .69

County-specific = .93

Disease-specific = .72

Vaccine-specific = .96

State Mandates = .75

Flu Vaccine = .94

Government Resources = .74

**Comments:**

Positive = .87

Neutral = .65

Negative = .78

Vaccine Safety = .74

Political Discussions = .76

Mask Wearing = N/A

State Mandates = .76

Vaccine Rollout = .72

Flu Vaccine = .90

Sheriff Enforcement = .66

Expressing Thanks = .98

## Coding Results

### **Table S8**

### Frequency of Emotional Valence and Topics in Posts by County-Level Death Rate

| **Variables** | **High Death Rate Counties,  N (%)** | **Low Death Rate Counties,  N (%)** | **Chi-squared, χ^2^(1,*N*=1588)** | ***p*-value** |
| --- | --- | --- | --- | --- |
| ***Emotional Valence*** | | |  |  |
| Positive | 52 (5.0%) | 53 (9.7%) | 12.91 | <.001 |
| Neutral | 974 (93.5%) | 487 (89.2%) | 8.92 | .003 |
| Negative | 15 (1.4%) | 4 (0.7%) | 1.52 | .331 |
| ***Topics*** | | |  |  |
| County-specific | 386 (37%) | 210 (38.5%) | .31 | .586 |
| Disease-specific | 93 (8.9%) | 21 (3.8%) | 13.87 | <.001 |
| Vaccine-specific | 740 (71.0%) | 348 (63.7%) | 8.80 | .004 |
| State Mandates | 68 (6.5%) | 22 (4.0%) | 4.12 | .040 |
| Flu Vaccine | 94 (9.0%) | 63 (11.5%) | 2.55 | .112 |
| Government Resources | 95 (9.1%) | 18 (3.3%) | 18.36 | <.001 |

*Note.* Coding categories were not mutually exclusive.

### **Table S9**

### Frequency of Emotional Valence and Topics in Posts by Political Party Affiliation

| **Variables** | **Republican-leaning Counties,  N (%)** | **Democrat-leaning Counties,  N (%)** | **Chi-squared, χ^2^(1,*N*=1588)** | ***p*-value** |
| --- | --- | --- | --- | --- |
| ***Emotional Valence*** | | |  |  |
| Positive | 37 (8.3%) | 68 (6.0%) | 2.79 | .115 |
| Neutral | 405 (90.6%) | 1056 (92.6%) | 1.65 | .217 |
| Negative | 2 (0.4%) | 17 (1.5%) | 2.95 | .121 |
| ***Topics*** | | |  |  |
| County-specific | 178 (39.8%) | 418 (36.6%) | 1.39 | .249 |
| Disease-specific | 19 (4.3%) | 95 (8.3%) | 8.00 | .005 |
| Vaccine-specific | 298 (66.7%) | 790 (69.2%) | .984 | .337 |
| State Mandates | 11 (2.5%) | 79 (6.9%) | 11.97 | <.001 |
| Flu Vaccine | 52 (11.6%) | 105 (9.2%) | 2.13 | .161 |
| Government Resources | 17 (3.8%) | 96 (8.4%) | 10.33 | .001 |

*Note.* Coding categories were not mutually exclusive.

### **Table S10**

### Frequency of Emotional Valence and Topics in Comments by County-Level Death Rate

| **Variables** | **High Death Rate Counties,  N (%)** | **Low Death Rate Counties,  N (%)** | **Chi-squared, χ^2^(1,*N*=1565)** | ***p*-value** |
| --- | --- | --- | --- | --- |
| ***Emotional Valence*** | | |  |  |
| Positive | 66 (8.1%) | 133 (17.8%) | 33.12 | <.001 |
| Neutral | 400 (49.0%) | 349 (46.7%) | .83 | .389 |
| Negative | 269 (32.9%) | 210 (28.1%) | 4.33 | .042 |
| ***Topics*** | | |  |  |
| Vaccine Safety | 61 (7.5%) | 73 (9.8%) | 2.62 | .124 |
| Political Discussions | 71 (8.7%) | 85 (11.4%) | 3.11 | .091 |
| Mask Wearing | 9 (1.1%) | 12 (1.6%) | .75 | .510 |
| State Mandates | 52 (6.4%) | 13 (1.7%) | 21.00 | <.001 |
| Vaccine Rollout | 66 (8.1%) | 57 (7.6%) | .11 | .778 |
| Flu Vaccine | 30 (3.7%) | 15 (2.0%) | 3.88 | .050 |
| Sheriff Enforcement | 11 (1.3%) | 9 (1.2%) | .063 | .826 |
| Expressing Thanks | 24 (2.9%) | 75 (10.0%) | 33.12 | <.001 |

*Note.* Coding categories were not mutually exclusive.

### **Table S11**

### Frequency of Emotional Valence and Topics in Comments by Political Party Affiliation

| **Variables** | **Republican-Leaning Counties,  N (%)** | **Democrat-Leaning Counties,  N (%)** | **Chi-squared, χ^2^(1,*N*=1565)** | ***p*-value** |
| --- | --- | --- | --- | --- |
| ***Emotional Valence*** | | |  |  |
| Positive | 87 (16.3%) | 112 (10.9%) | 9.34 | .003 |
| Neutral | 259 (48.5%) | 490 (47.5%) | .134 | .749 |
| Negative | 147 (27.5%) | 332 (32.2%) | 3.62 | .064 |
| ***Topics*** | | |  |  |
| Vaccine Safety | 51 (9.6%) | 83 (8.1%) | 1.01 | .341 |
| Political Discussions | 72 (13.5%) | 84 (8.1%) | 11.16 | <.001 |
| Mask Wearing | 8 (1.5%) | 13 (1.3%) | .150 | .817 |
| State Mandates | 7 (1.3%) | 58 (5.6%) | 16.45 | <.001 |
| Vaccine Rollout | 47 (8.8%) | 76 (7.4%) | .99 | .323 |
| Flu Vaccine | 12 (2.2%) | 33 (3.2%) | 1.15 | .340 |
| Sheriff Enforcement | 9 (1.7%) | 11 (1.1%) | 1.07 | .344 |
| Expressing Thanks | 55 (10.3%) | 44 (4.3%) | 21.6 | <.001 |

*Note.* Coding categories were not mutually exclusive.
